# Clinical outcomes of laparoscopic-assisted synchronous bowel anastomoses for synchronous colorectal cancer: initial clinical experience

**DOI:** 10.18632/oncotarget.12899

**Published:** 2016-11-03

**Authors:** Zhengtian Li, Dawei Wang, Yunwei Wei, Peng Liu, Jun Xu

**Affiliations:** ^1^ Department of General Surgery, The First Affiliated Hospital of Harbin Medical University, Harbin, China; ^2^ Department of Colorectal Surgery, Changhai Hospital, Second Military Medical University, Shanghai, China

**Keywords:** colorectal surgery, laparoscopy, synchronous bowel anastomoses, synchronous colorectal cancer

## Abstract

The primary aim of this study was to explore the safety and feasibility of laparoscopic-assisted synchronous bowel anastomoses (LSBA) for synchronous colorectal cancer (SCRC). All patients who underwent LSBA for SCRC were retrospectively reviewed and analyzed for clinical and pathological features, technical feasibility and short-term as well as long-term oncological outcomes. Between July 2008 and January 2012, a series of 11 consecutive SCRC patients underwent LSBA. Six patients underwent laparoscopic-assisted right hemicolectomy and anterior resection. Five patients had laparoscopic-assisted right hemicolectomy and sigmoidectomy. There were no intraoperative complications that required open conversions. Mean operation time was 233 (range, 195–285) minutes, and mean estimated blood loss was 224 (range, 100–300) mL. The postoperative course of the patients was uneventful with the mean return to oral intake was 6.9 (range 5–12) days, and mean length of hospital stay was 12.6 (range 9–17) days. All surgical wounds showed good cosmetic outcome, and the mean incision length was 4.1 (range 3.5-5.0) cm. During a median follow-up period of 76 months, no local tumor recurrences were found. LSBA is a potentially feasible and safe procedure for SCRC when performed by an experienced surgeon. Further large clinical controlled trials are warranted to confirm the findings.

## INTRODUCTION

Synchronous colorectal cancer (SCRC) is defined as more than one primary cancer in the colorectum at the time of resection or within six months, accouting for 1.1-8% of all primary colorectal cancer [1–5]. Surgical resection is the first treatment option. However, there still remains much controversy regarding what is the best treatment option for synchronous lesions in multiple surgical segments [4, 6–9]. For some selected patients, synchronous bowel anastomoses without fecal diversion can be achieved with good results [10, 11].

Widespread use of laparoscopy in the past two decades has achieved a tremendous impact on colorectal surgery among surgical community for its advantages over open approaches in short and long-term outcomes [12–14]. With the rapid technical innovations and the concept of natural orifice surgery (NOTES) and single incision laparoscopic surgery (SILS) put forward in minimal invasive surgery, surgeons are pursuing some more challenging complex colorectal procedures and gaining good results in terms of minimal pain, scarless, lower costs, and faster recovery and similar morbidity rate [14–27].

However, the role of laparoscopy in SCRC with multiple segmental resections and synchronous bowel anastomoses (LSBA) is unclear and rarely reported in that surgeons lack randomized studies or clear evidence regarding the risks with synchronous bowel anastomoses. Such trials can only be found in some case reports [28–33]. This study was designed to initially assess the feasibility and short-time outcomes with LSBA for patients with SCRC. The second aim of our study is to systematically review the literature regarding this procedure.

## RESULTS

There were totally eight male and three female patients with a mean age of 63.5 (range 51–81) years, body mass index of 21.1 (range 17.8–23.5) kg/m^2^. Detailed patient characteristics are presented in Table 1. The tumor location was in ascending colon and sigmoid colon (n=4), cecum and upper rectum (n=2), ascending colon and upper rectum (n=4) and cecum and sigmoid colon (n=1). Surgical procedures included six laparoscopic-assisted right hemicolectomy and anterior resection and five laparoscopic-assisted right hemicolectomy and sigmoidectomy. Each procedure involved two anastomoses. There were no intraoperative complications that required open conversions. Mean operation time was 233 (range, 195–285) minutes, and mean estimated blood loss was 224 (range, 100–300) mL. The postoperative course of most patients was uneventful with the mean return to oral intake was 6.9 (range 5–12) days, and mean length of hospital stay was 12.6 (range 9–17) days. Three patients experienced some mild morbidity (one with mild ileus symptoms, one with urinary retention and the other with wound liquefaction) who all recovered with conservative management. All surgical wounds showed good cosmetic outcome, and the mean incision length was 4.1 (range 3.5-5.0) cm. During a median follow-up period of 76 months, no local tumor recurrences occured.

**Table 1:** Patient characteristics and surgical outcomes

Case	Sex	Age	ASA	BMI	Tumors location	Operation procedure	Incision length (cm)	Operative time (min)	Estimated blood loss (ml)
1	F	62	2	22.9	Ascending colon + upper rectum	RHC+AR	4.5	220	300
2	M	81	3	21.9	Cecum + upper rectum	RHC+AR	4.5	215	200
3	M	53	2	19.2	Ascending colon + sigmoid colon	RHC+SC	5.0	200	100
4	M	76	2	22.3	Ascending colon + sigmoid colon	RHC+SC	4.0	267	300
5	M	68	2	23.5	Ascending colon + sigmoid colon	RHC+SC	3.5	255	180
6	F	54	1	20.4	Ascending colon + upper rectum	RHC+AR	4.0	210	220
7	M	60	2	22.6	Cecum + sigmoid colon	RHC+SC	4.0	265	250
8	F	71	3	19.7	Ascending colon + upper rectum	RHC+AR	3.5	195	200
9	M	51	2	20.7	Cecum + upper rectum	RHC+AR	4.5	285	240
10	M	66	2	17.8	Ascending colon + upper rectum	RHC+AR	3.5	235	190
11	M	56	1	21.3	Ascending colon + sigmoid colon	RHC+SC	4.5	220	280

Abbreviations: ASA, American Society of Anesthesiologists; BMI, body mass index; RHC, right hemicolectomy; SC, sigmoidectomy; AR, anterior resection.

Pathological results included 22 tumors with nine adenomatous canceration, eleven moderately differentiated adenocarcinoma and two adenocarcinoma mixed with mucinous carcinoma. The mean size of resected tumors was 3.7 (range, 2.5–5.5) cm. The mean number of harvested lymph nodes was 17 (range, 11–25) and three patients (27%) were found to have lymph node metastasis on pathological examination. The surgical margins were all negative, and the mean incision length was 4.1 (range 3.5-5.0) cm.

**Figure 1:**
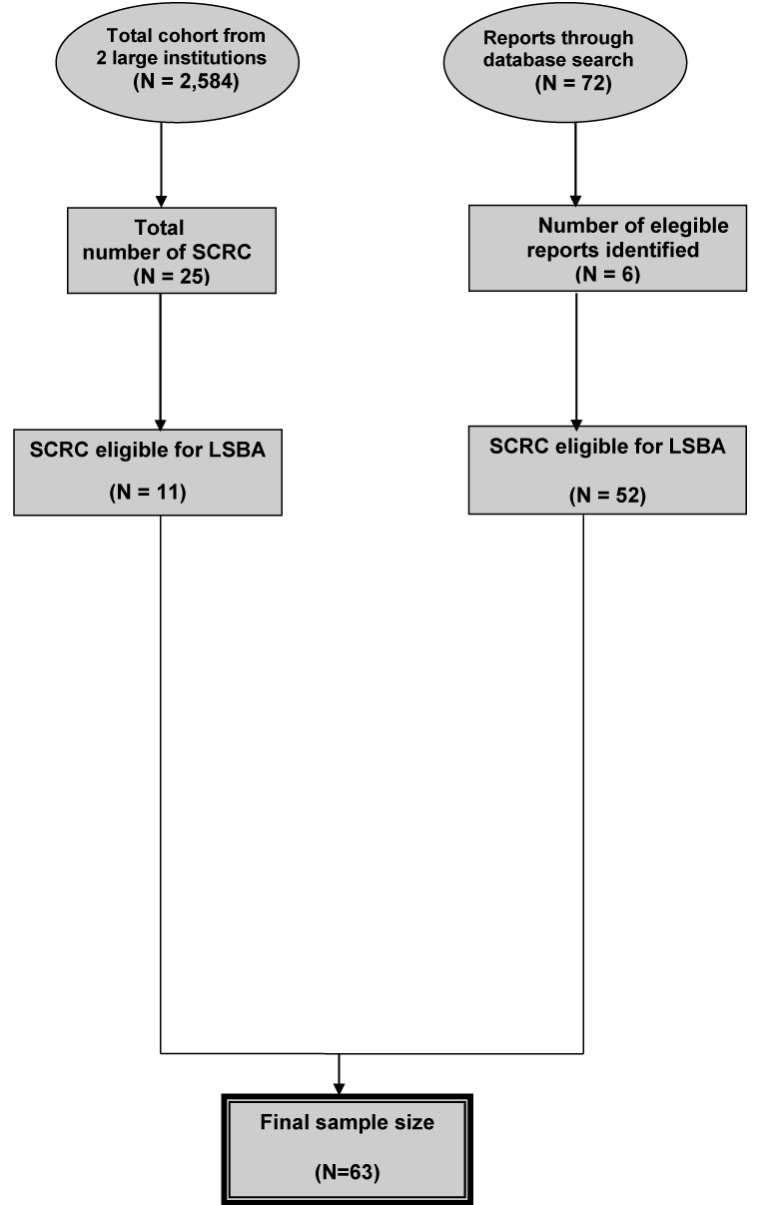
Flow chart of case selection

We conducted a review of the literature, which yielded a total of 72 citations from the initial search. After full text review, six reports totaling 52 SCRC cases operated with LSBA [28–33] met the inclusion criteria (Figure 1). The sample size ranged from one to 27. No patient experienced conversion to laparotomy. As is shown in Table 3, most of the included patients gained favarable clinical and cosmetic outcomes. Three studies reported eight comodities including two with enterocolitis, three cases with surgical site infection, two with ileus and one with anastomotic leak. No operative mortality was reported. However, these studies did not report long term oncologic results.

**Table 2:** Pathological data and postoperative outcomes

Case	RH pathology				SC or AR pathology				Bowel movement (day)	Time of liquid diet intake (day)	Hospital stay (day)	Morbidity
	Size	T	LN-T No.retrieved	LN-P No.positive	Size	T	LN-T No.retrieved	LN-P No.positive				
1	5.0	2	16	0	4.0	2	14	0	5	6	10	None
2	5.0	3	11	0	3.5	2	13	0	10	12	17	Mild ileus
3	5.5	3	25	3	4.5	3	22	1	4	5	9	None
4	4.5	3	16	0	3.0	3	11	0	6	7	13	None
5	2.5	2	16	0	2.5	1	19	0	5	7	12	None
6	3.0	1	15	0	4.5	3	17	3	3	5	10	None
7	3.5	2	11	0	3.5	2	18	0	6	7	14	None
8	3.0	2	16	0	3.0	3	20	0	7	9	17	Urinary retention
9	4.5	3	15	1	3.5	2	24	0	4	5	11	None
10	3.5	2	20	0	3.0	1	18	0	6	7	14	Wound liquefaction
11	2.5	1	13	0	4.0	2	16	0	4	6	12	None

Abbreviations: T, T staging; RH, right hemicolectomy; SC, sigmoidectomy; AR, anterior resection; LN-T No.retrieved, total number of lymph nodes retrieved; LN-P No.positive, number of metastatic lymph nodes.

**Table 3:** Summary of case series reporting laparoscopic-assisted synchronous bowel anastomoses for synchronous colorectal cancer

Author, reference	Sample size	Age (ys)	Weight or BMI	Tumor location	Operation procedure	Incision length (cm)	Operative time (min)	Estimated blood loss (ml)	Postoperative Discharge day	Morbidity
Lauter, 200329	2	NR	NR	Right colon + low rectum	RH+LAR	Maximun:6	220	NR	Both on day 3	None
Jafari, 200728	1	85	73 kg	Hepatic flexure + low rectum	RH+LAR(with colonic J-pouch anal anastomosis)	4	185	Minimal	Day 3	None
Tan, 201233	1	70	22.0 kg/m2	Ascending colon +upper rectum	RH+AR	6	175	Minimal	NR	None
Inada, 201431	11	Median 71	Median 23.3 kg/m2	Right-sided colon+left-sided col on	RH+LH	Median5	Median 296	Median: 65	Most on day 8	2 with surgical site infection.
Fang, 201532	10	Mean 61.3	16.6-27.6 kg/m2	Right or left colon+rectum	RH/LH +LAR	Mean 4	Mean 198	Mean 73	Mean on day 10	1 with incision infection
Takatsu, 201530	27	Median 66	Mean 21.9 kg/m2	Right or transverse colon+rectum or sigmoid	RH/T+AR/SR	Mean 5.2	Mean 373	Mean 40	Mean on day 12	2 with enterocolitis, 2 with ileus and 1 with anastomotic leak

Abbreviations: BMI, body mass index; LAR, low anterior resection; LH, left hemicolectomy; NR, not reported; RH, righ hemicolectomy; SC, sigmoidectomy; T, partial resection of the transverse colon.

## DISCUSSION

Laparoscopic-assisted surgical intervention for some selected complicated colorectal disease has evolved considerably during the past few years, with a more frequent trend to replace open procedure. However, less data are available regarding the proper role of laparoscopic surgery for some patients with SCRC who need to have multiple segmental resections and synchronous bowel anastomoses. This preliminary study represents one of the first case series to use the approach of LSBA.

For low prevalence of SCRC, there is no common consensus on its operative strategies and extents of resection [34]. Moreover, one of the disadvantages of laparoscopic approach is its lack of tactile sensation, the limitation of visualization and some small inconspicuous lesions in the abdominal cavity or bowel may sometimes be overlooked. So careful preoperative evaluation (routine abdominal computed tomography with contrast enema, total colonoscopy and positron emission tomography) should be emphasized to avoid or prevent missed diagnosis. The surgical options and extents of resection are determined by several factors, which is demonstrated in previous literature [10]. We consider it a comprehensive understanding of a patient's general situation including nutritional status, staging of the lesions, intraoperative situation, technical availability and the factor of the surgeon himself. In our study, the evaluation method mentioned above indicated no lymph node metastasis or distal metastasis, so we did not perform extensive resections. Nor did we prefer a subtotal colectomy or total proctocolectomy in that the elderly patients had less physiologic reserve to tolerate such a major operation. Such an option may have important consequences on the reserve absorptive capacity of the colon, which may largely improve the patients’ quality of life and functional status. And laparoscopic approach has its own advantages over open procedure as it provides decreased surgical trauma, fewer perioperative complications, and faster postoperative recovery with similar survival rates [12, 13]..

Due to the existence of two anastomoses simutaneously, the potential risk of anastomotic dehiscence and other complications seems reasonably to be higher. However, no major complications were seen within 30 days after operation in our study, except for one with mild ileus symptoms who recoved with conservative management. Moreover, the shorter incision greatly reduced the wound-related morbidity associated with conventional open colorectal procedures which require a long midline incision, usually extended from the xiphoid process to the symphysis pubis, contributing to a more prolonged period of postoperative pain, analgesic consumption and poorer cosmetic result, and even adding to the risk of abdominal incisional hernia. Though we performed the operations in two different bowel segments, both specimens in one procedure were retrieved through one small incision as planned.

The primary aim of the current study is to determine whether LSBA for SCRC can be safely performed with acceptable outcomes. And it does show the feasibility without major perioperative complications if we refine selection criteria. In our study, the patients had no obesity-associated conditions (mean body mass index, 21 kg/m^2^) or history of abdominal or pelvic surgery. The relatively small size of the tumors (mean size, 3.7cm) and thin mesenteric tissues faciliate the extraction of specimens. Moreover, adequate preoperative bowel preparation and good nutritional condition may be a high priority for reducing the incidence of complications. We consider it of great importance to eliminate hard feces in the bowel in case that they may be confused with lesions for lack of tactile sensation by the laparoscopic surgeons.

Despite the small sample size, this study demonstrates that an LSBA approach can be utilized for some more challenging colorectal surgeries and achieves similar good clinical and cosmetic results to the previous ones. Its operative times with LSBA may be no longer and estimated blood loss less than those reported with an open approach [10]. However, comparative study and further assessment will be made for the limitations of a small sample size. Furthermore, we obtain favarable long-term oncological results without local recurrence during the follow-up period, which has not been reported in the previous studies.

To the best of our knowledge, the major strength of this study is that this is the first case series that has assessed not only the short-term outcomes but also the long-term oncological outcome of LASB. However, limitations to our study should be addressed. The small sample size did not allow us to draw definite conclusions regarding the true outcomes for this technique. In addition, the study did not have control group, which limited the interpretation of the true effect for LASB. Even so, most of the previously studies also only reported short-term outcome without long-term oncological results. Further large-scale, well-designed, randomized controlled studies are warranted to confirm these findings.

In summary, LSBA seems to be a feasible and safe procedure when performed by an experienced surgeon. It may be an alternative technique for the treatment of some selected patients in short-term outcomes with SCRC. However, additional large clinical controlled trials should be advocated to evaluate the safety and efficacy as well as oncological outcomes of LSBA for the treatment of SCRC in the future.

## MATERIALS AND METHODS

### Patient data

This study was based on a retrospective review of a prospectively maintained database in the Department of Colorectal Surgery at two large institutions between July 2008 and January 2012, which included a total of 2584 consecutive colorectal cancer patients. Twenty-five patients were diagnosed as SCRC, from which a series of 11 patients were selected to undergo LSBA. Patient demographics (such as age, gender, and body mass index), clinical data (such as tumor location, operation procedure, and incision length), pathologic and postoperative outcomes (such as tumor size, number of retrieved lymph node, and time of bowel movement or liquid diet intake) were collected based on medical record review. This study was approved by the Ethical Committee of the First Affiliated Hospital of Harbin Medical University and Changhai Hospital, which was carried out in accordance with the approved guidelines. The informed consent was obtained from all the investigated subjects.

### Indication for LSBA

In our study, LSBA was generally indicated for SCRC with two tumors distributed in different surgical segments. All lesions were localized through total colonoscopy with titanium clips, barium enema and computed tomography preoperatively. Intraoperative colonoscopy was performed during resection if necessary. Preoperative evaluation indicated there was no evident lymph node metastasis or evident invasion to the adjacent organs. Final diagnosis was established through colonoscopy and pathology. We excluded the following patients: (1) those with locally advanced tumor who were treated by neoadjuvant chemotherapy, (2) those who were inability to general anesthesia or pneumoperitoneum, (3) those with intestinal obstruction and mechanical bowel preparation could not be routinely done.

### Operative technique

LSBA procedures were performed by the surgeons who had already completed the learning curve with experience of more than 200 colorectal procedures and acquired sufficient experience in laparoscopic colorectal surgery. We followed the oncological principles including en bloc resection with complete lymphadenectomy, no-touch technique, proximal lymph-vascular ligation, wound protection, adequate resected margin of the colon and total mesorectal excision for rectal cancer. The ileostomy site is marked preoperatively in the lower right quadrant by an enterostomal therapist. Under general anesthesia, the patient was placed in a Trendelenburg tilt position with legs abducted. A cushion was placed beneath the pelvis for adequate exposure of the anorectal region. CO2 pneumoperitoneum was established via a Veress needle and maintained at an endoabdominal pressure of 10 to 12 mmHg. Five trocars ranging from 5–12 mm (subumbilical; right lower quadrant; right upper quadrant; left lower quadrant; left upper quadrant; suprapubic) and a 30° angled laparoscopy were used in our approach. An additional suprapubic trocar was added as assistance if necessary.

For sigmoid colon cancer or upper rectal cancer, we routinely mobilized the splenic flexure for tension-free anastomosis. The inferior mesenteric artery (IMA) and inferior mesenteric vein (IMV) were ligated near the origin of IMA with three laparoscopic Hem-o-lok clips (Weck Closure System, Research Triangle Park, NC), respectively. After mobilization the splenic flexure and distal bowel to rectosigmoid junction for sigmoid colon cancer or to rectum at least 5cm from the tumor's inferior pole for upper rectum cancer, we transected the distal edge with one or more Endo GIA (Auto Suture) under laparoscopic guidance. Hypogastric nerves, left ureter and gonadal vessels were clearly identified and better preserved.

Then right hemicolectomy proceeded. After locating the tumor, we used a medial-to-lateral approach. The extent of the resection depended on the location of the tumor. In our four cases, as the tumors were all close to the ileocecum, we only divided the right branch of the middle colic artery with the main trunk preserved. The ileocolic artery and right colic artery were ligated at their origins. After the major lymphovascular pedicles had been divided and ligated, the right colon along with its mesentery and greater omentum were fully mobilized. Both specimens were retrieved through a 4cm extension of a subumbilical incision covered with a plastic wound protector.

For right-sided colonic lesion, after resection of the specimen, a stapled end-to-side ileotransverse colon anastomosis was created extracorporeally. Then the bowel was returned to the abdomen. And for sigmoid or upper rectal lesion, the proximal bowel was divided and an anvil was inserted into the proximal colon which was also returned to the abdomen for a standard end-to-end anastomosis intracorporeally with circular stapler (Proximate CDH29; Johnson & Johnson) under laparoscopic visualization. Each anastomosis was ensured tension-free with a good blood supply. A drainage tube with negative pressure was placed near each anastomosis through one of the trocar sites. No diverting ileostomies were created. Finally, every specimen was carefully examined before the abdominal trocar wound was closed.

### Literature review

To identify relevant publications regarding surgical treatment of SCRC, we searched the Pubmed and EMBASE databases by using the following keywords: colorectal/colonic/rectal/ gastrointestinal neoplasms; neoplasms, multiple primary and laparoscopy. Additionally, manual reference search of relevant publications was also performed. We did not limit date or language to the search strategy. We selected, identified relevant studies and extracted data by two independent reviewers. Discrepancies were resolved by all the reviewers.
